# Pig Milk Exosome Packaging ssc-miR-22-3p Alleviates Pig Intestinal Epithelial Cell Injury and Inflammatory Response by Targeting *MAPK14*

**DOI:** 10.3390/ijms251910715

**Published:** 2024-10-05

**Authors:** Jie Li, Huihui Hu, Panpan Fu, Qiaoli Yang, Pengfei Wang, Xiaoli Gao, Jiaojiao Yang, Shuangbao Gun, Xiaoyu Huang

**Affiliations:** 1 College of Animal Science and Technology, Gansu Agricultural University, Lanzhou 730070, China; lijie5272@126.com (J.L.);; 2 Gansu Research Center for Swine Production Engineering and Technology, Lanzhou 730070, China

**Keywords:** pig milk exosomes, ssc-miR-22-3p, cell viability and proliferation, intestinal inflammatory injury

## Abstract

Inflammatory diseases of the intestinal tract in piglets severely impair the economic performance of pig farms. Pig milk exosomes can encapsulate miRNAs which can then enter the piglet intestine to play an immunomodulatory role. Previously, we comparatively analyzed and identified exosomal miRNAs in the colostrum and mature milk of Bamei and Landrace pigs, and we screened for ssc-miR-22-3p, which is associated with inflammation and immune response; however, the role played by ssc-miR-22-3p in the immune response in IPEC-J2 cells is not yet clear. In this study, we first constructed a pig intestinal inflammatory response model using Lipopolysaccharide (LPS) and Polyinosinic-polycytidylic acid (Poly (I:C)), and we investigated the role of ssc-miR-22-3p targeting *MAPK14* in the regulation of LPS and Poly (I:C)-induced inflammatory injury in IPEC-J2 cells by RT-qPCR, cell counting kit-8 (CCK-8), EdU staining, lactate dehydrogenase (LDH) activity assay, and dual luciferase reporter gene assay. We successfully established LPS and Poly (I:C)-induced cell damage models in IPEC-J2 cells. The immune response of IPEC-J2 cells was stimulated by induction of IPEC-J2 cells at 10 μg/mL LPS and 20 μg/mL Poly (I:C) for 24 h. Overexpression of ssc-miR-22-3p decreased cytokine expression and promoted cell viability and proliferation. The functional enrichment analysis revealed that ssc-miR-22-3p targets genes enriched in the pathways of negative regulation of inflammatory response and bacterial invasion of epithelial cells. The validity of the binding site of ssc-miR-22-3p to *MAPK14* was tested by a dual luciferase reporter gene. Pig milk exosome ssc-miR-22-3p promotes cell viability and proliferation by targeting *MAPK14*, and it alleviates LPS and Poly (I:C)-induced inflammatory responses in IPEC-J2 cells.

## 1. Introduction

In recent years, piglet intestinal inflammatory disease has been widely prevalent, which is prone to causing intestinal dysfunction in piglets, which directly affects the feed conversion rate and growth of piglets, and even causes piglet deaths. Due to the intermittent nature of the outbreaks, which reoccur easily, the economic benefits of pig farms are seriously undermined. Bacteria and viruses are the main pathogens causing diarrhea and enteric diseases in piglets. After invading, pathogens can increase intestinal epithelial permeability [1], activate the immune signal transduction pathway, induce the production of pro-inflammatory cytokines, and damage tissue oxidation [2], and this eventually leads to intestinal inflammation and other diseases, such as impaired intestinal barrier function, inflammation, diarrhea, and immune response [3]. In particular, newborn piglets are more susceptible to infection. Therefore, it is of great importance to study the mechanism of intestinal inflammatory response that is regulated by molecular disease resistance.

Milk serves as a vital source of nutrients for piglets post-birth and a crucial avenue for acquiring passive immunity. It encompasses a range of nutrients, including carbohydrates, proteins, fats, and bioactive substances that function as natural anti-infective and anti-inflammatory agents in breast milk [4]. These substances also constitute significant components of the innate immune system [5]. An increase in breast milk has been shown to reduce the morbidity and mortality associated with inflammatory diseases [6,7]. Beyond its nutrient-rich content, milk-derived exosomes represent one of the biologically active substances with intricate secretion mechanisms. They contain a diverse array of protein, fat, and nucleic acid components [8]. Research has revealed that these exosomes perform various biological functions, such as facilitating cell communication, promoting cell proliferation, immunoregulation, and signaling between immune cells. Notably, they carry immune-related structures that enhance the development and maturation of both the immune system and intestines [9]. When nursing, piglets can absorb exosomes through the intestinal tract [10], which deliver proteins and nucleic acids to receptor cells by vesicular transport, thereby regulating intestinal inflammatory and immune responses [11,12]. Consequently, milk exosomes play pivotal roles in the development and barrier function of the intestines. They may contribute significantly to the prevention and treatment of intestinal inflammatory diseases [13].

MicroRNAs (miRNAs) occupy pivotal positions in a diverse array of physiological and pathological processes encompassing growth and development, differentiation, proliferation, and immune responses [14,15]. These small non-coding RNAs regulate mRNA expression and translation by targeting the 3ʹ UTR of specific genes through complementary sequences, thereby inhibiting their expression and translation [16,17]. Previous research has demonstrated that pig milk exosomes harbor an abundant repertoire of miRNAs [18], and these exosome-encapsulated miRNAs are intimately linked to intestinal pathophysiological processes [11]. Notably, studies have shown that pig milk exosomal miRNAs play a crucial role in modulating Deoxynivalenol (DON)-induced intestinal epithelial cell damage [19]. Furthermore, exosomal miR-207 has been found to alleviate stress symptoms in mice [20]. The transfer of exosomal miRNAs to offspring through milk and its role in the development of the immune system have garnered considerable attention from scholars globally.

Lipopolysaccharide (LPS), a cytotoxic endotoxin derived from the cell wall of Gram-negative bacteria, is known to disrupt intestinal morphology and barrier function, and it is frequently employed in the establishment of intestinal inflammatory response models [21,22]. Polyinosinic-polycytidylic acid (Poly (I:C)) is a synthetic double-stranded RNA mimicking viral components, and it is commonly used to simulate pathogen infections [23,24]. Specifically, LPS and Poly (I:C) models are utilized to mimic bacterial and viral infections, respectively [23], and they are well-established models of bacterial and double-stranded RNA viral infections [25]. Therefore, we utilized LPS and Poly (I:C) to simulate the infection model in the two cases, and we compared and explored the role of ssc-miR-22-3p in the two infection modes.

In our previous research, we conducted a preliminary screening of various miRNAs present in the milk exosomes of Bamei and Landrace pigs through miRNA-seq sequencing, identifying a subset of differentially expressed miRNAs [26]. Furthermore, we performed GO and KEGG enrichment analyses on these differentially expressed genes and selected immune-related miRNAs (ssc-miR-340, ssc-miR-21-5p, and ssc-miR-22-3p) from signature immune-related signaling pathways. Next, we reviewed the existing literature on ssc-miR-340, ssc-miR-21-5p, and ssc-miR-22-3p and found that ssc-miR-22-3p exhibits potent immune effects. These findings sparked our interest in investigating the immunomodulatory role of ssc-miR-22-3p in porcine milk exosomes. Therefore, our further study on the molecular mechanism of ssc-miR-22-3p in IPEC-J2 cells’ resistance to LPS and Poly (I:C)-induced intestinal epithelial cell damage will provide important scientific evidence for exploring the regulation of milk exosomal miRNAs on intestinal inflammatory responses.

## 2. Results

### 2.1. Effect of LPS and Poly(I:C) on IPEC-J2 Cell Activity and Cytokine Expression

**Figure 1:**
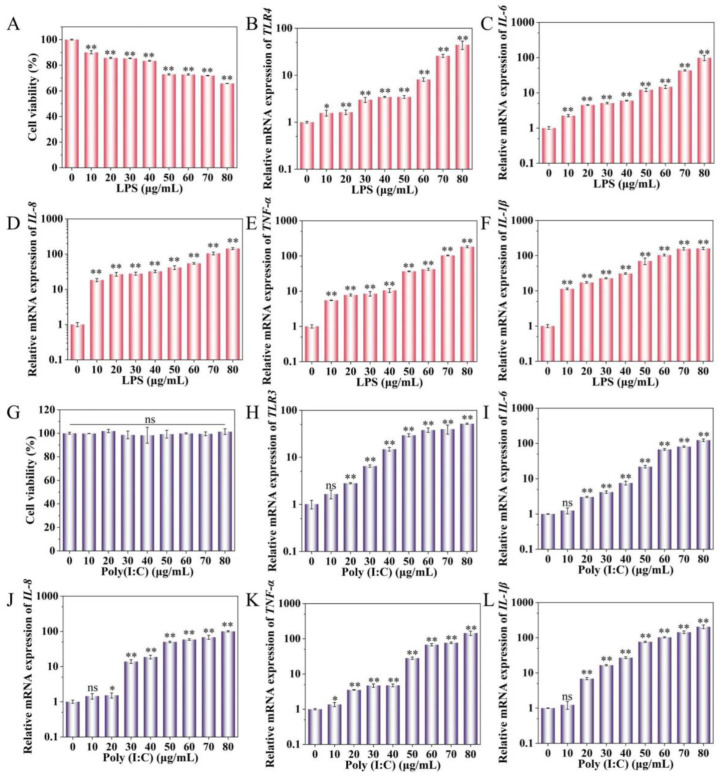
Effects of LPS and Poly(I:C) on IPEC-J2 cell viability and cytokine expression. (**A**) Effects of different concentrations of LPS on IPEC-J2 viability. (**B**–**F**) Effects of different concentrations of LPS on IPEC-J2 *TLR4*, *IL-6*, *IL-8*, and *TNF-α* mRNA expression, respectively. (**G**) Effects of different concentrations of Poly(I:C) on IPEC-J2 viability. (**H**–**L**) Effect of different concentrations of Poly(I:C) on IPEC-J2 *TLR3*, *IL-6*, *IL-8*, and *TNF-α* mRNA expression, respectively. * *p* < 0.05, ** *p* < 0.01, ns indicates no significant difference.

The viability of IPEC-J2 cells was assessed following treatment with various concentrations of LPS (10–80 μg/mL) for a duration of 24 h. The results indicated that a significant reduction in cell viability occurred with 10 μg/mL LPS treatment compared to the control group (*p* < 0.01). Furthermore, as the concentration of LPS increased, cell viability decreased progressively (Figure 1A). In addition, upon stimulating IPEC-J2 cells with different concentrations of LPS, there was a marked upregulation in the expression of *TLR4*, *IL-6*, *IL-8*, *TNF-α*, and *IL-1β* genes. All these changes were statistically significant compared to the control group (*p* < 0.05) (Figure 1B–F).

Exposure of IPEC-J2 cells to various concentrations of Poly (I:C) (ranging from 10 to 80 μg/mL) for 24 h had no discernible effect on cell viability (Figure 1G). Furthermore, when IPEC-J2 cells were treated with 10 μg/mL of Poly (I:C) for 24 h, only *TNF-α* exhibited a significant upregulation (*p* < 0.05), while the expression levels of other cytokines remained unchanged (Figure 1K). Notably, upon stimulation with Poly(I:C) concentrations of 20–80 μg/mL, the expression levels of *TLR3*, *IL-6*, *IL-8*, *TNF-α*, and *IL-1β* were significantly elevated compared to the control group (*p* < 0.05) (Figure 1H–L). These findings indicate that IPEC-J2 cells mounted an immune response when treated with LPS and Poly (I:C) at concentrations of 10 and 20 μg/mL, respectively, for 24 h. Consequently, we have successfully established cell damage models in IPEC-J2 cells induced by LPS and Poly (I:C).

### 2.2. Expression Levels of ssc-miR-22-3p in LPS and Poly(I:C)-Stimulated IPEC-J2 Cells

**Figure 2:**
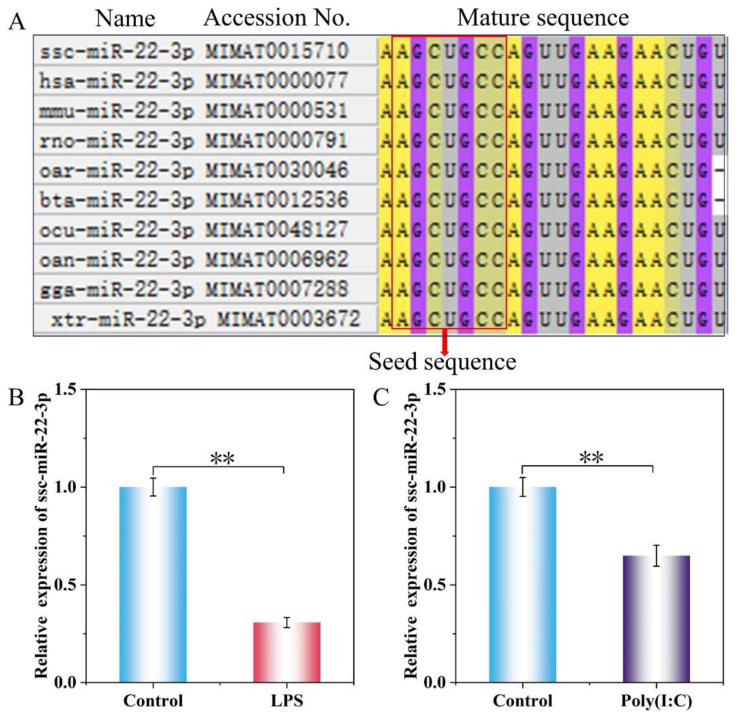
The relative expression levels of ssc-miR-22-3p in LPS and Poly (I:C)-induced IPEC-J2 cells. (**A**) Sequence conservation analysis of miR-22-3p matrices from different species. (**B**,**C**) Relative expression levels of ssc-miR-22-3p after stimulation of IPEC-J2 cells with LPS and Poly (I:C), respectively. ** *p* < 0.01. The red arrow indicates the seed sequence.

The homology of miR-22-3p mature sequences among ten species—including *Sus scrofa* (ssc), *Homo sapiens* (hsa), *Mus musculus* (mmu), *Rattus norvegicus* (rno), *Ovis aries* (oar), *Bos taurus* (bta), *Oryctolagus cuniculus* (ocu), *Omithorhynchus anatinus* (oan), *Gallus galluscuniculus* (gga), and *Xenopus tropicalis* (xtr)—was analyzed by MEGA11.0 software. The analysis revealed a high degree of homology for miR-22-3p across these species, with identical sequences in the seed region located at positions 2 to 8 at the 5′ end (Figure 2A).

After the IPEC-J2 cells were treated with LPS (10 μg/mL) and Poly (I:C) (20 μg/mL) for 24 h, the expression of ssc-miR-22-3p was significantly lower than that of the control group in both the LPS and Poly groups (*p* < 0.01) (Figure 2B,C), which was hypothesized to possibly play a regulatory role in the LPS and Poly (I:C)-induced inflammation response of IPEC-J2 cells. However, this regulatory function needs to be further confirmed through additional experiments.

### 2.3. ssc-miR-22-3p Transfection Efficiency Assay

**Figure 3:**
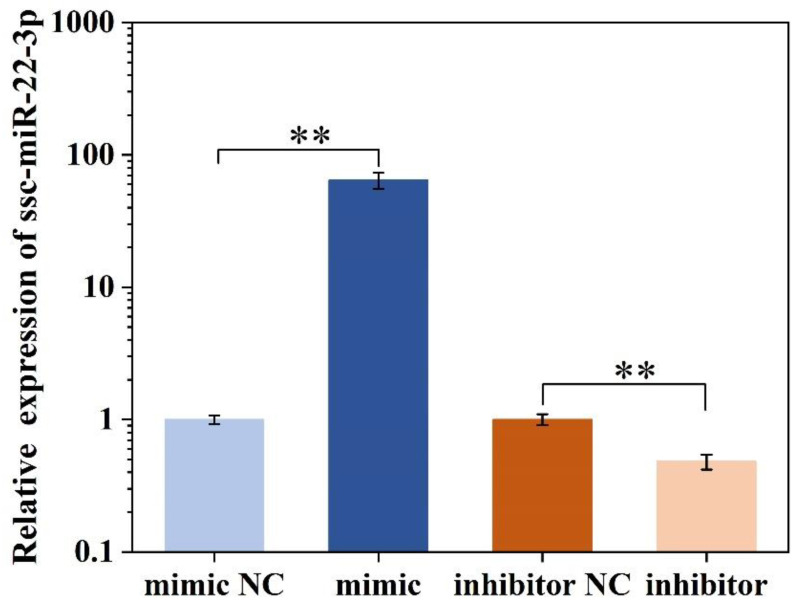
ssc-miR-22-3p transfection efficiency assay. ** *p* < 0.01.

IPEC-J2 cells were transfected with either a miR-22-3p mimic or its negative control (mimic NC), as well as with a miR-22-3p inhibitor or its negative control (inhibitor NC). Following the 24 h transfection period, the expression levels of miR-22-3p were assessed using RT-qPCR. When compared to cells transfected with mimic NC, those transfected with the miR-22-3p mimic exhibited a significant upregulation of miR-22-3p expression (*p* < 0.01; Figure 3). Conversely, cells transfected with the miR-22-3p inhibitor showed significant downregulation of miR-22-3p expression compared to those transfected with inhibitor NC (*p* < 0.01; Figure 3). These results indicate that the miR-22-3p mimic and inhibitor demonstrated high overexpression and knockdown efficiencies, respectively, and are suitable for use in subsequent experiments.

### 2.4. Upregulation of ssc-miR-22-3p Significantly Inhibits LPS and Poly (I:C)-Induced IPEC-J2 Cell Cytokine Expression

**Figure 4:**
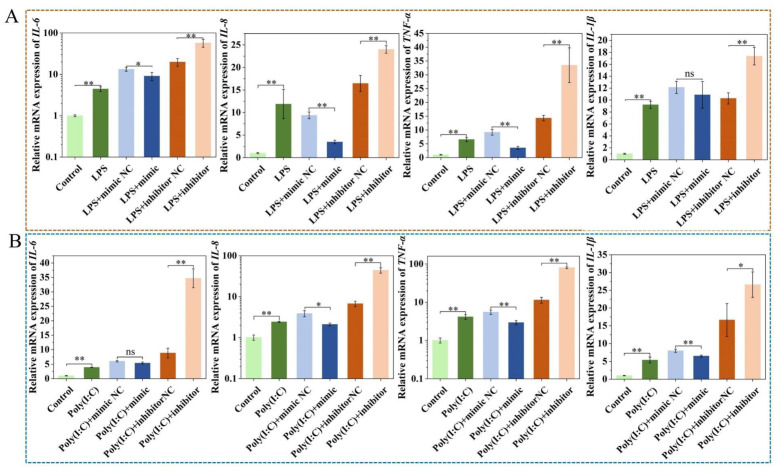
Effect of ssc-miR-22-3p on the effect of LPS (**A**) and Poly (I:C) (**B**)-induced inflammatory responses in IPEC-J2 cells. * *p* < 0.05, ** *p* < 0.01, ns means no significant difference.

After transfection with the miR-22-3p mimic, the expression of inflammatory cytokines *IL-6*, *IL-8*, *TNF-α*, and *IL-1β* were differentially downregulated in IPEC-J2 cells treated with LPS and Poly (I:C). Conversely, the inhibitor resulted in varying degrees of upregulation of these inflammatory cytokines (*p* < 0.05) (Figure 4A,B). These results indicated that miR-22-3p mimic alleviated cellular injury and inflamma-tory response.

### 2.5. Effect of ssc-miR-22-3p on LPS and Poly (I:C)-Induced IPEC-J2 Cell Cytotoxicity

**Figure 5:**
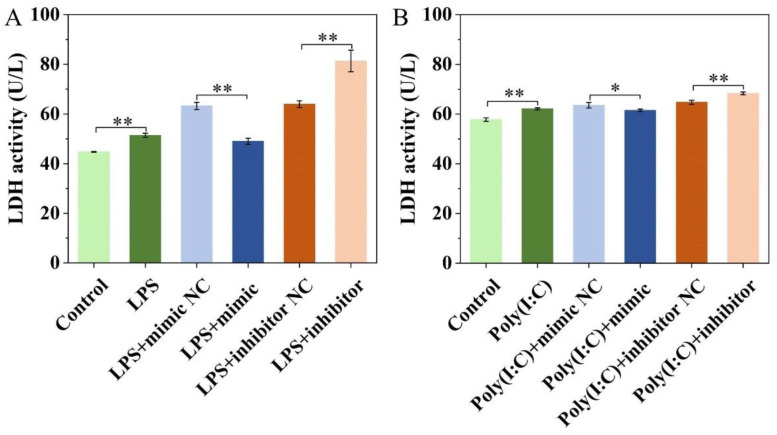
Effect of ssc-miR-22-3p on LPS (**A**) and Poly (I:C) (**B**)-induced IPEC-J2 cell cytotoxicity. * *p* < 0.05, ** *p* < 0.01.

After transfecting IPEC-J2 cells treated with LPS and Poly (I:C) with the miR-22-3p mimic, a significant reduction in LDH activity was observed in the cell supernatant (*p* < 0.05). In contrast, transfection with the miR-22-3p inhibitor led to a significant increase in LDH activity (*p* < 0.01) (Figure 5A,B). These results indicate that overexpression of ssc-miR-22-3p decreases cytotoxicity.

### 2.6. Effect of ssc-miR-22-3p on LPS and Poly (I:C)-Induced IPEC-J2 Cell Viability and Proliferation

**Figure 6:**
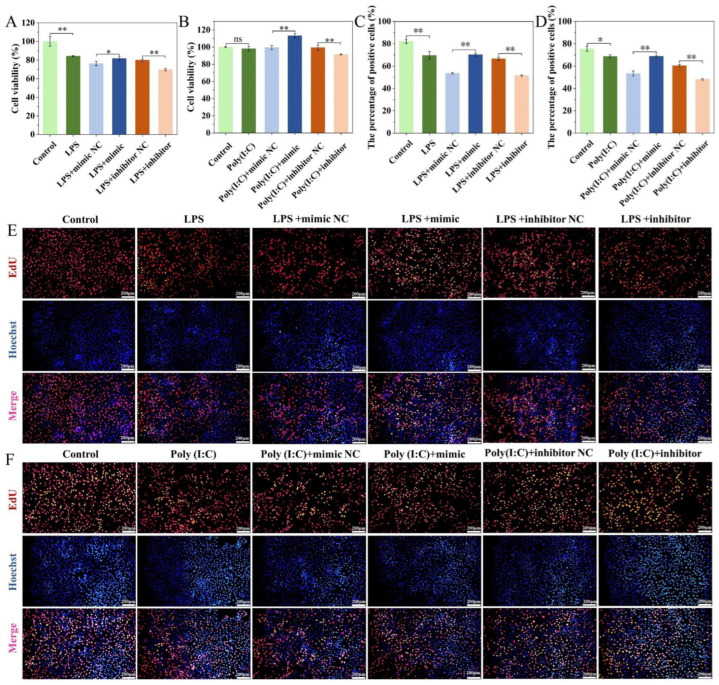
Analysis of ssc-miR-22-3p on LPS and Poly (I:C)-induced IPEC-J2 cell viability and proliferation. (**A**,**B**) indicates the effect of ssc-miR-22-3p on the viability of LPS and Poly (I:C)-induced IPEC-J2 cells, respectively. (**C**,**D**) indicates the effect of ssc-miR-22-3p on the number of LPS and Poly (I:C)-induced IPEC-J2 positive cells, respectively. (**E**,**F**) EdU cell proliferation assay. Scale bar = 200 µm. * *p* < 0.05, ** *p* < 0.01, ns indicates no significant difference.

Treatment with LPS resulted in a reduction in IPEC-J2 cell viability, whereas Poly (I:C) had no significant impact on cell viability. When compared to their respective negative controls, transfection with the miR-22-3p mimic significantly enhanced IPEC-J2 cell viability (*p* < 0.05). Conversely, transfection with the miR-22-3p inhibitor led to a significant decrease in cell viability (*p* < 0.05) (Figure 6A,B).

The results of the EdU staining assay demonstrated (Figure 6C–F) that both LPS and Poly (I:C) treatments led to a reduction in EdU-positive signals and inhibited cell proliferation compared to the control group. When compared to the miR-22-3p mimic negative control (NC), the number of EdU-positive cells in the group transfected with the miR-22-3p mimic was significantly increased (*p* < 0.05), indicating a promotion of cell proliferation. In contrast, the number of EdU-positive cells was significantly decreased in the group transfected with the miR-22-3p inhibitor (*p* < 0.01). These findings suggest that overexpression of ssc-miR-22-3p promotes cell proliferation and viability in IPEC-J2 cells treated with LPS and Poly (I:C).

### 2.7. Target Gene and Functional Enrichment Analysis of ssc-miR-22-3p

**Figure 7:**
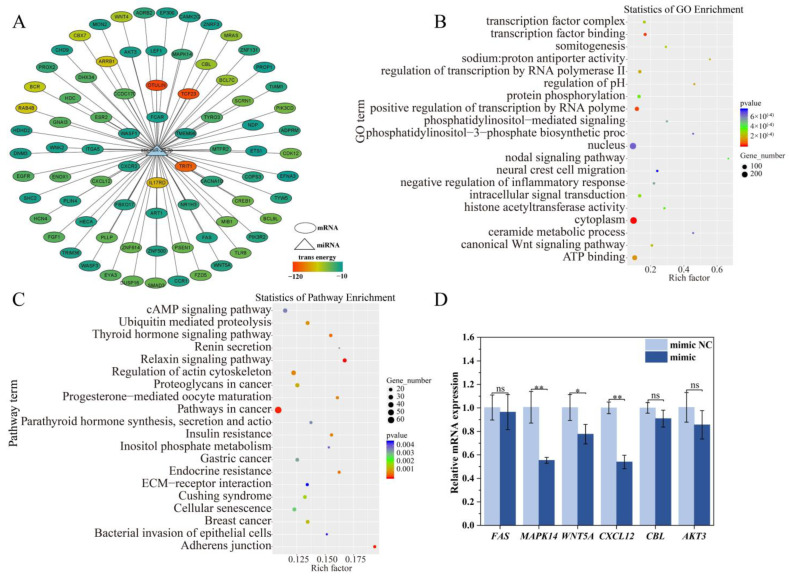
Functional enrichment analysis of ssc-miR-22-3p. (**A**) ssc-miR-22-3p and mRNA gene interaction network diagram. The ellipse represents mRNA, the triangle represents miRNA, and the color of the ellipse represents the size of the free energy for miRNA to form with the mRNA sequence. (**B**,**C**) represents GO and KEGG enrichment analysis of ssc-miR-22-3p target genes, respectively. (**D**) RT-qPCR verification of immune-related target genes. * *p* < 0.05, ** *p* < 0.01, ns indicates no significant difference.

To further investigate the function of ssc-miR-22-3p, we conducted a series of analyses. First, we predicted target genes and constructed target gene interaction networks. Next, we performed functional enrichment analysis and identified genes related to the immune response, including *FAS*, *MAPK14*, *WNT5A*, *CXCL12*, *CBL*, and *AKT3* (Figure 7A). The significantly enriched Gene Ontology (GO) terms included the canonical Wnt signaling pathway, intracellular signal transduction, and negative regulation of the inflammatory response (Figure 7B). Additionally, a Kyoto Encyclopedia of Genes and Genomes (KEGG) enrichment analysis revealed significant enrichment in pathways related to cancer, breast cancer, cAMP signaling, and bacterial invasion of epithelial cells (Figure 7C).

To validate the accuracy of the predicted target gene results, RT-qPCR was conducted to assess the mRNA expression levels of six crucial target genes, as depicted in Figure 7D. The findings revealed that, following transfection with the ssc-miR-22-3p mimic, the expression levels of the target genes *MAPK14*, *WNT5A*, and *CXCL12* were notably downregulated compared to the control group *(p* < 0.05). Although the expressions of *FAS*, CBL, and AKT3 also exhibited a downward trend, these changes were not statistically significant (*p* > 0.05). Based on these observations, we hypothesize that ssc-miR-22-3p may specifically regulate the *MAPK14*, *WNT5A*, and *CXCL12* genes, thereby modulating the LPS and Poly(I:C)-induced inflammatory damage in IPEC-J2 cells.

### 2.8. Validation of ssc-miR-22-3p and MAPK14 Targeting Relationship

**Figure 8:**
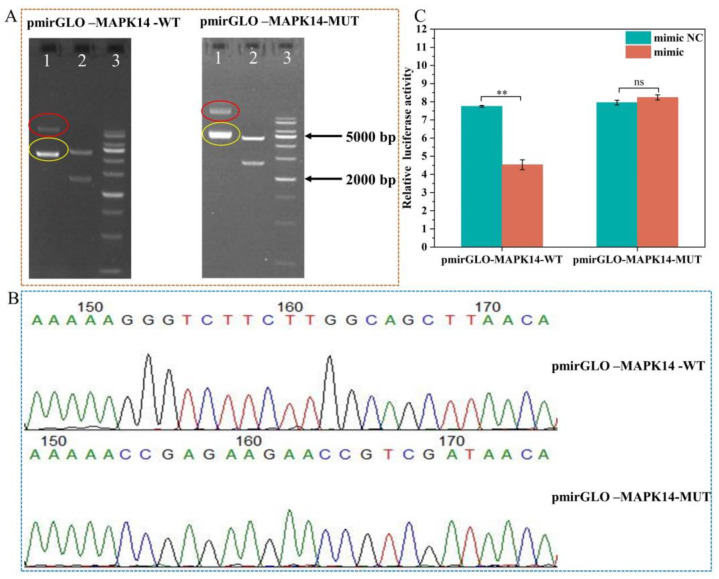
Identification of the validity of the targeting relationship between ssc-miR-22-3p and *MAPK14*. (**A**) *MAPK14* recombinant vector enzyme cleavage map. Lane 1: red circle indicates self-labeled empty vector before digestion, yellow indicates recombinant vector before digestion; Lane 2: vector and target fragment after digestion with HindIII; Lane 3: 1 kb ladder. (**B**) Plot of Sanger sequencing peaks of pmirGLO-*MAPK14*-WT and pmirGLO-*MAPK14*-MUT. (**C**) The targeting relationship between ssc-miR-22-3p and *MAPK14* was detected by dual luciferase reporter gene. ** *p* < 0.01, ns indicates no significant difference.

To further confirm the targeting relationship between ssc-miR-22-3p and its negatively regulated target genes, *MAPK14* was randomly selected for dual luciferase reporter experiments. We constructed the recombinant vectors pmirGLO-*MAPK14*-WT and pmirGLO-*MAPK14*-MUT. The amplified products were verified by 1% agarose gel electrophoresis, and the recombinant vectors were further confirmed through double digestion with restriction endonucleases NheI and XhoI. The results indicated that the size of the target fragment matched the expected size (Figure 8A), confirming the successful construction of the recombinant vector for the 3′ UTR of the *MAPK14* gene. Using the constructed recombinant vector as a template, specific primers were designed for the amplification and sequencing of the target fragment. Sequencing analysis confirmed the successful mutation of the binding site (Figure 8B), paving the way for subsequent dual luciferase activity detection experiments.

Next, HEK 293 T cells were transfected with recombinant plasmid and miRNA mimics/inhibitors for 48 h. A dual luciferase reporting system was used to verify whether ssc-miR-22-3p binds to the 3′ UTR of *MAPK14*. The luciferase activity of the co-transfected group with pmirGLO-*MAPK14*-WT and the miR-22-3p mimic was significantly lower than that of the co-transfected group with pmirGLO-*MAPK14*-WT and the mimic NC (*p* < 0.01). There was no statistically significant difference in luciferase activity between the pmirGLO-*MAPK14*-MUT and miR-22-3p mimic co-transfection group and the pmirGLO-*MAPK14*-MUT and mimic NC co-transfection group (*p* > 0.05) (Figure 8C). The results of the dual luciferase reporting system showed that the ssc-miR-22-3p mimics are involved in the regulation of *MAPK14* expression by binding to a predetermined binding site in the 3′ UTR.

## 3. Discussion

Intestinal inflammatory injury-induced immune dysfunction poses a significant threat to the health and growth of piglets. Addressing the alleviation of intestinal diseases in piglets resulting from such inflammatory injury is crucial for improving the economic efficiency of pig farming. The intestinal tract serves as the primary barrier for the body’s immune response, with the small intestinal mucosa and epithelial cells being the primary targets for pathogenic bacterial invasion. Pathogenic bacterial infections activate immune signaling pathways, leading to the production of pro-inflammatory cytokines and oxidative tissue damage [2,27].

Exosomal miRNAs play pivotal roles in crucial biological processes, including immune regulation within the body [28]. Notably, breast-milk-derived exosomal miRNAs associated with immunity exhibit robust stability and resistance to degradation [29]. These miRNAs can be transmitted to offspring through breastfeeding and contribute to the development of their immune systems [30]. Furthermore, breast-milk-derived exosomes have been shown to protect intestinal epithelial from oxidative-stress-induced cell death [31]. Research has indicated that exosomes from rat milk can enhance intestinal epithelial cell viability, promote proliferation, and stimulate intestinal stem cell activity [32]. Similarly, pig milk exosomes have been found to promote intestinal epithelial cell proliferation [33]. Based on these findings, we hypothesized that pig-milk-exosome-derived ssc-miR-22-3p is involved in inflammatory responses and regulates cell proliferation. To this end, we investigated the mechanism by which pig milk exosome ssc-miR-22-3p modulates LPS and Poly (I:C)-induced inflammatory responses in IPEC-J2 cells.

First, we established inflammatory models for bacterial and viral infections and conducted a comparative analysis of the distinct pro-inflammatory mechanisms triggered by these two stimuli. Upon stimulating IPEC-J2 cells with LPS and Poly (I:C), we observed an increase in the release of *TLR4*, *TLR3*, *IL-6*, *IL-8*, *TNF-α*, and *IL-1β*, thereby successfully establishing the inflammatory models. LPS and Poly (I:C) infections stimulate the secretion of pro-inflammatory cytokines, which are pivotal in initiating the activation of the immune response. Specifically, LPS stimulates *TLR4*, leading to the release of pro-inflammatory cytokines in the immune response [34]. Toll-like receptors (TLRs) constitute a significant class of innate immune recognition molecules, with *TLR4* and *TLR3* serving as the recognition receptors for LPS and Poly (I:C), respectively, and are indispensable for the innate immune response [23,35]. Inflammatory responses mediated by TLRs through the LPS/TLR4 signaling pathway enhance the production of pro-inflammatory cytokines such as *IL-1β*, *IL-6*, and *TNF-α* [36]. Following infection with Poly (I:C), the ligand for macrophage TLR3 becomes activated [37]. Similarly, Poly (I:C) induces an upregulation of TLR3 expression in dendritic cells [38]. In our study, LPS induced an increase in cytokine release and a decrease in cell viability, aligning with the findings of numerous other studies. A study revealed that intestinal epithelial cells exposed to LPS exhibited a significant increase in inflammatory cytokine release while simultaneously experiencing suppressed cell viability [39]. In addition, our findings indicated that Poly (I:C) also led to an elevation in cytokine release but did not significantly impact cell viability. A possible explanation for this phenomenon is that, although Poly(I:C) can elicit immune activation, it may not directly trigger the signaling pathways that lead to cell death. Another plausible explanation is that the immune activation induced by Poly(I:C) may actually exert a protective effect on cells. Poly (I:C) induced murine mammary carcinoma cells and fibrosarcoma cells had no significant effect on cell viability and induced upregulation of pro-inflammatory cytokines (*IL-6*, *TNFα*) and chemokines (*CXCL10*) [40]. Similarly, Poly (I:C) induced elevated levels of inflammatory cytokines in human keratinocytes and macrophages [41,42]. The levels of pro-inflammatory cytokines *CXCL10*, *CXCL1*, *CCL2*, *CCL5*, *IL-1β*, *IL-6*, and *TNF-α* were higher in plasma and bronchoalveolar lavage in the Poly (I:C)-constructed mouse injury model [23,43].

MiRNAs are highly conserved across the majority of animal species and play a pivotal role [44,45]. Ssc-miR-22-3p demonstrates a high level of mature sequence conservation among various species, hinting at its potential to fulfill similar biological functions. To determine the effect of ssc-miR-22-3p on LPS- and Poly (I:C)-induced inflammatory responses in IPEC-J2 cells, we examined the expression of ssc-miR-22-3p in an injury model and found that the expression of ssc-miR-22-3p was significantly downregulated, which was hypothesized to be potentially involved in the regulation of inflammatory responses.

Pig milk exosomal miRNAs can alleviate intestinal inflammation by inhibiting the release of inflammatory factors and apoptosis of intestinal epithelial cells. The milk-derived exosome ssc-miR-22-3p promotes the proliferation of human intestinal epithelial cells (HIEC) by targeting and inhibiting the expression of CCAAT/enhancer-binding protein δ (C/EBPδ) [46]. The miR-22-3p overexpression inhibited LPS-induced increases in *IL-1β*, *IL-6*, *TNF-α*, and *NO* to alleviate LPS-induced inflammation and apoptosis in HK-2 cells by targeting *PTEN* [47]. Furthermore, miR-22-3p can also inhibit cell proliferation, although this may be due to species specificity in other cells or animals [48]. The highly expressed miRNAs (miR-181a, miR-30c, miR-365-5p, and miR-769-3p) in pig milk exosomes significantly reduced the protein expression levels of the p53 pathway target genes, and they ultimately attenuated Deoxynivalenol (DON)-induced IPEC-J2 cell injury by promoting cell proliferation and inhibiting apoptosis [19]. Deng et al. [49] found that miR-590-3p, an exosome secreted by M2 cell macrophages, could alleviate intestinal inflammation by inhibiting LPS-induced release of pro-inflammatory cytokines *TNF-α*, *IL-1β*, and IL-6 from FHC cells through modulation of the LATS1/YAP/ β-Catenin axis. In our study, ssc-miR-22-3p inhibited LPS- and Poly (I:C)-induced inflammatory responses in IPEC-J2 cells, promoted cell proliferation and increased cell viability, and alleviated intestinal inflammatory injury, which is similar to the above findings. Of course, miR-22-3p also has potential side effects. miR-22-3p inhibits the expression of target genes by binding to the 3′ UTR of their mRNA. Abnormal expression levels of miR-22-3p may lead to excessive inhibition or insufficiency of target gene expression, thereby triggering a series of disruptions in biological functions. This imbalance can affect the normal processes of cell proliferation, differentiation, and apoptosis, ultimately contributing to the occurrence or progression of diseases.

The milk-derived exosome ssc-miR-22-3p is believed to play a pivotal role in modulating the immune response of intestinal epithelial cells [46]. In this study, functional enrichment analysis of the target gene set of ssc-miR-22-3p showed that its targets were significantly enriched in the canonical Wnt signaling pathway, as well as the negative regulation of inflammatory response and bacterial invasion of epithelial cells signaling pathways. These pathways are intricately linked to inflammatory responses and immune regulation. Notably, the canonical Wnt signaling pathway itself is closely associated with immune cell function regulation [50] and can exacerbate the pro-inflammatory response phenotype of macrophages [51]. Previous studies have highlighted the Wnt/β-catenin signaling pathway’s essential role in regulating proliferation, differentiation, and apoptosis [52]. Furthermore, this pathway has emerged as a promising therapeutic target for bacterial infectious diseases, as it controls the expression of various genes that regulate the inflammatory response in infections induced by pathogenic bacteria [53]. In the regulatory processes of organisms, multiple signaling cascades often interact and contribute to the complex regulation of inflammation. Besides the aforementioned signaling pathways, there are potential involvements of other signaling pathways, such as the cAMP and breast cancer signaling pathways. Thus, we hypothesized that ssc-miR-22-3p might be involved in the invasion of IPEC-J2 cells by LPS and Poly (I:C) by regulating target genes.

Proteins in an organism function through interactions. Protein–Protein Interaction (PPI) analyses are important for studying most biological functions and processes [54]. In order to study the target genes of ssc-miR-22-3p, we screened out genes (*FAS*, *MAPK14*, *WNT5A*, *CXCL12*, *CBL*, and *AKT3*) associated with immune response by PPI network analysis combined with GO and KEGG enrichment analysis. Further, PT-qPCR was used to screen out genes (*MAPK14*, *WNT5A*, and *CXCL12*) that have negative regulatory relationships with ssc-miR-22-3p, which play a crucial role in the intestinal inflammatory response. There are many studies reporting that *MAPK14* acts as a signaling regulator to modulate the biosynthesis of cytokines (*TNF-α*, *IL-1*, *IL-6*, and *IL-1β*), which can induce the release of proteins associated with inflammatory responses and is often used as a therapeutic target for inflammatory diseases [55,56]. *WNT5A* is involved in the regulation of physiological processes such as cell motility, proliferation, and differentiation [57], which can activate the non-canonical Wnt signaling pathway to activate the pro-inflammatory signaling cascade and increase the secretion of pro-inflammatory cytokines and chemokines [58]. *WNT5A*, because of the presence of two protein isoforms, has different functions; it exerts both anti-inflammatory and pro-inflammatory effects in inflammatory diseases, and it may be a potential diagnostic marker for inflammatory diseases [59]. In addition, *WNT5A* significantly reduces LPS-induced cytokine and chemokine production [60]. *CXCL12*, an anti-inflammatory chemokine in autoimmune diseases, is involved in a variety of inflammatory responses [61,62], and it blocks and attenuates inflammatory responses [63].

MiRNAs predicted by bioinformatics software miRanda (v3.3a) and TargetScan (v5.0) have false positives, and verification by dual luciferase reporter genes is more powerful evidence to determine the authenticity of miRNA and mRNA binding sites [64]. Therefore, to verify whether ssc-miR-22-3p interacts with its target genes to regulate the inflammatory response of pig intestinal epithelial cells, we further performed dual luciferase reporter gene experiments. In our study, overexpression of ssc-miR-22-3p significantly inhibited *MAPK14* wild-type luciferase reporter gene activity, and no significant difference was observed in the mutant plasmid, indicating that the binding site between ssc-miR-22-3p and *MAPK14* is real and effective. The above studies suggest that miRNAs in exosomes can target key mRNAs in intestinal-inflammation-related signaling pathways, thereby affecting the release of inflammatory factors and modulating intestinal inflammatory responses. However, our experiment investigated the short-term impact of ssc-miR-22-3p targeting *MAPK14* on IPEC-J2 cells in vitro under the condition of LPS and Poly (I:C) challenge. We did not study the long-term effects of ssc-miR-22-3p on porcine intestinal health or survival, which, indeed, represents a limitation of our work. In the future, we will investigate the long-term impact of ssc-miR-22-3p on regulating porcine intestinal health or survival in vivo, filling the current knowledge gap and providing new insights for the development of safe and effective miRNA-based strategies to improve porcine intestinal health.

## 4. Materials and Methods

### 4.1. Cell Culture, Transfection, and Construction of Cell Injury Models

Pig small intestinal epithelial cells (IPEC-J2 cells) were acquired from the BeNa Culture Collection in Beijing, China, and maintained in Dulbecco’s Modified Eagle Medium (DMEM) supplemented with 10% fetal bovine serum (FBS; Gibco, Waltham, MA, USA) and 1% penicillin/streptomycin (Hyclone, Logan, UT, USA). The cells were incubated at 37 °C with 5% CO_2_. Upon reaching 80% confluence, the cells were trypsinized with 0.25% Trypsin-EDTA (Gibco, Waltham, MA, USA) and plated into 24-well plates. For transfection, Lipofectamine™ Reagent 2000 (Invitrogen, Waltham, MA, USA) was used according to the manufacturer’s instructions.

The IPEC-J2 cells were exposed to varying concentrations (10, 20, 30, 40, 50, 60, 70, and 80 μg/mL) of LPS (*Escherichia coli* (055:B5), Sigma-Aldrich, Shanghai, China) and Poly (I:C) (Invitrogen, Carlsbad, CA, USA) for 24 h to establish a cell injury model. Subsequently, the optimal dosage for treatment was determined by evaluating cell survival and inflammatory cytokine expression levels, guiding the selection of LPS and Poly (I:C) concentrations for subsequent functional validation.

### 4.2. Lactate Dehydrogenase (LDH) Activity Assay

The cells were transfected and treated with toxin for 24 h. Following this, the cell culture medium was harvested and centrifuged at 4000 rpm for 5 min to obtain the supernatant. The LDH concentration in the supernatant was then assayed using an LDH Assay Kit (Jancheng Bioengineering Institute, Nanjing, China), with the absorbance read at 450 nm as measured by an enzyme counter.

### 4.3. Cell Viability and Proliferation Assays

Cell viability was assessed using the cell counting kit-8 (CCK-8) assay. The IPEC-J2 cells were transfected with either an overexpression or interfering plasmid for ssc-miR-22-3p for 24 h, followed by a 24 h stimulation with LPS and Poly (I:C). Subsequently, 10 μL of CCK-8 Solution (Beyotime, Shanghai, China) was added to each well and incubated in a cell culture incubator for 4 h. The absorbance at 450 nm was then measured using an enzyme marker.

For the proliferation analysis, the transfected IPEC-J2 cells, which had also been exposed to LPS and Poly (I:C), were incubated with EdU solution for 2 h, as per the instructions provided with the BeyoClick™ EdU Cell Proliferation Kit with Alexa Fluor 555 (Beyotime, Shanghai, China). The cells were then subjected to an EdU staining assay, and the positive proliferating cells were observed using an Olympus IX71 fluorescence microscope (Olympus IX71, Tokyo, Japan).

### 4.4. Target Gene Prediction and Bioinformatics Analysis of ssc-miR-22-3p

To gain deeper insights into the functional mechanism of ssc-miR-22-3p, we utilized the OmicStudio platform (https://www.omicstudio.cn/tool/7) (accessed on 24 April 2024), which integrates the TargetScan (v5.0) [65,66,67] and miRanda (v3.3a) [68,69,70] algorithms. By identifying the overlapping genes predicted by both software tools, we predicted the target genes of ssc-miR-22-3p. The prediction was further refined using specific thresholds: TargetScan_score ≥ 50 and miranda_Energy < −10 as the screening thresholds. Gene Ontology (GO) and Kyoto Encyclopedia of Genes and Genomes (KEGG) pathway enrichment analyses were performed using DAVID (https://david.ncifcrf.gov/) (accessed on 27 April 2024) software for enrichment analysis. The interaction network of target genes regulated by ssc-miR-22-3p was visualized through the application of Cytoscape (V3.8.0). Additionally, the homology analysis of *Sus scrofa* with the mature sequences of miR-22-3p from other species was executed using MEGA 11.0 software.

### 4.5. Screening of Immune-Related Target Genes

In this study, we simultaneously utilized both TargetScan (v5.0) and miRanda (v3.3a) software to predict target genes, resulting in the identification of 1608 common target genes through intersection analysis. We then analyzed the binding sites based on the target gene interaction network, focusing on both the matching score (TargetScan_score) and the free energy (miranda_Energy). To further explore functional enrichment, we initially screened six key target genes (*FAS*, *MAPK14*, *WNT5A*, *CXCL12*, *CBL*, and *AKT3*) in conjunction with inflammation and immune-associated GO and KEGG-enriched pathways. Next, we transfected IPEC-J2 cells with miR-22-3p mimics and mimic NC, and we used RT-qPCR to assay the impact of ssc-miR-22-3p on the expression of these genes. We subsequently identified genes with negative regulatory relationships as potential target genes. Finally, we randomly selected *MAPK14* for validation using a dual luciferase reporter gene assay.

### 4.6. Construction of Wild-Type and Mutant Plasmids in the MAPK14 3′ UTR Region

**Figure 9:**
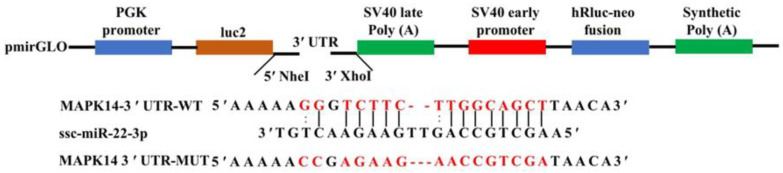
Schematic diagram of pmirGLO-*MAPK14*-3′ UTR recombinant vector construction.

The *MAPK14* (XM_001929490.6) gene sequence was downloaded from the National Center for Biotechnology Information (NCBI; https://www.ncbi.nlm.nih.gov/) (accessed on 10 May 2024) repository. Both the wild type and mutant sequences within the 3ʹ UTR region of the *MAPK14* gene were synthesized and subsequently inserted into the dual luciferase reporter gene vector, named pmirGLO. The cloning sites utilized for this insertion were 5′ NheI and 3′ XhoI (Figure 9). In order to construct the *MAPK14* 3ʹ UTR double luciferase reporter gene wild-type vector (pmirGLO-*MAPK14*-WT) and mutant vector (pmirGLO-*MAPK14*-MUT), the vector construction was synthesized by Genewiz Biotechnology Co., Ltd. (Jiangsu, China). Both miRNA overexpression and interfering plasmids were designed and synthesized by GenePharma (Shanghai, China), and their sequences are shown in Table 1.

### 4.7. Dual Luciferase Reporter Gene Activity Assay

HEK-293T cells were cultivated in DMEM supplemented with 10% FBS and placed in an incubator at 37 °C with 5% CO_2_. Cells exhibiting robust growth were seeded into 24-well plates. Transfection was carried out following the protocol provided by Lipofectamine^®^ Reagent 2000 (Invitrogen, Carlsbad, CA, USA) and in accordance with the miRNA mimic instructions. Specifically, HEK-293T cells were co-transfected with either a negative control mimic (mimic NC) or a miR-22-3p mimic, along with either the *MAPK14*-3′ UTR wild-type or mutant recombinant plasmids. Four experimental groups were established: pmirGLO-*MAPK14*-WT co-transfected with mimic NC, pmirGLO-*MAPK14*-WT co-transfected with miR-22-3p mimic, pmirGLO-*MAPK14*-Mut co-transfected with mimic NC, and pmirGLO-*MAPK14*-Mut co-transfected with miR-22-3p mimic. Forty-eight hours post-transfection, the HEK-293T cells were subjected to a luciferase reporter gene assay. The activities of firefly luciferase and sea kidney luciferase were determined in 96-well enzyme-labeled plates, adhering to the manufacturer’s instructions of the Dual Luciferase Reporter Assay Kit (Vazyme, Nanjing, China). The relative luciferase activity was calculated as the ratio of the firefly luciferase fluorescence value to the sea kidney luciferase fluorescence value. Each treatment was evaluated with three biological replicates.

### 4.8. Real-Time Quantitative Polymerase Chain Reaction (RT-qPCR)

Total RNA was extracted utilizing Trizol reagent. Subsequently, cDNA for mRNA quantification was synthesized in accordance with the protocol provided by the Evo M-MLV RT Mix Kit with gDNA Clean for qPCR Ver.2 (Accurate Biotechnology, Hunan, China). Similarly, cDNA for miRNA quantification was synthesized by following the instructions outlined in the miRNA First strand cDNA synthesis kit (Accurate Biotechnology, Hunan, China). RT-qPCR was performed using SYBR^®^ Green Premix *Pro Taq* HS qPCR Kit and LightCycler 480 Instrument II (Roche, Basel, Switzerland). The RT-qPCR reaction conditions comprised an initial pre-denaturation step at 95 °C for 30 s, followed by 40 cycles of denaturation at 95 °C for 5 s and annealing at 60 °C for 30 s. Each sample was assayed in triplicate, and the relative expression was calculated by the 2^−∆∆Ct^ method [71].

The mature sequence of ssc-miR-22-3p was retrieved from the miRBase database (https://www.mirbase.org/) (accessed on 8 April 2024). Based on this sequence, specific upstream primers were designed, while a miRNA qPCR 3ʹ primer was utilized for downstream amplification. For normalization purposes, the *U6* gene served as the internal reference gene. Meanwhile, mRNA sequences were sourced from the NCBI and downloaded accordingly. These sequences were then used to design primers with the aid of Prime-Blast software (https://www.ncbi.nlm.nih.gov/) (accessed on 12 April 2024). *GAPDH* was chosen as the mRNA reference gene, and all primers were synthesized by Genewiz Biotechnology Co., Ltd. (Suzhou, China). The detailed specifications of the RT-qPCR primers are shown in Table 2.

### 4.9. Statistical Analyses

The experimental data were statistically analyzed using IBM SPSS 25 (SPSS, Chicago, IL, USA). Significance analysis was performed using the Student’s *t*-test (two-tailed test), and the data were expressed as mean ± SD. Graphs were plotted using Origin 2018 (OriginLab, Waltham, MA, USA) software; * *p* < 0.05 for a significant difference, ** *p* < 0.01 for an extremely significant difference, and ns for no significant difference.

## 5. Conclusions

Pig milk exosome packaging ssc-miR-22-3p alleviates LPS and Poly (I:C)-induced IPEC-J2 inflammatory responses and promotes cell proliferation and viability by targeting *MAPK14*. This study lays the foundation for the study of milk exosome miRNA regulation of intestinal inflammatory response. However, we did not assess the long-term impact of ssc-miR-22-3p on regulating porcine intestinal health or survival in vivo, which, indeed, represents a limitation of our work.

## Figures and Tables

**Table 1:** MiRNA mimic and inhibitor sequence information.

Name	Sense (5′–3′)	Antisense (5′~3′)
mimic NC	UUCUCCGAACGUGUCACGUTT	ACGUGACACGUUCGGAGAATT
miR-22-3p mimic	AAGCUGCCAGUUGAAGAACUGU	AGUUCUUCAACUGGCAGCUUUU
inhibitor NC	CAGUACUUUUGUGUAGUACAA	
miR-22-3p inhibitor	ACAGUUCUUCAACUGGCAGCUU	

**Table 2:** RT-qPCR primers to amplify specific gene sequences.

Gene Name	Nucleotide Sequence (5ʹ-3ʹ)	Product Length (bp)
*FAS*	F: CGTGAGGGTCAATTCTGCTGTC	134
	R: GAATGATGGTTCTTGTCTGTGTAATCC	
*MAPK14*	F: TTATCTCATTAACAGGATGCCAAGCC	127
	R: CTCCAGCAAGTCAACAGCCAAG	
*WNT5A*	F: TCTTGGTGGTCCTTAGGTATGAATAAC	135
	R: GTGGTCCTGATACAAGTGGCATAG	
*CXCL12*	F: ATGCCCTTGCCGATTCTTTGAG	126
	R: CACACTTGTCTATTGTTGCTCTTCAG	
*CBL*	F: GGACAAGAAGATGGTGGAGAAGTG	143
	R: AGATAGTGCGGAGATGCTGGTAG	
*AKT3*	F: TTCTCTGGAGTAAACTGGCAAGATG	135
	R: AGGTGGCGTTATTGTAATAGTCTGAG	
ssc-miR-22-3p	F: CGCAAGCTGCCAGTTGAAGAACT	
	R: miRNA qPCR 3ʹ primer (Universal primers)	
*U6*	F: GGAACGATACAGAGAAGATTAGC	68
	R: TGGAACGCTTCACGAATTTGCG	
*GAPDH*	F: AGTATGATTCCACCCACGGC	139
	R: TACGTAGCACCAGCATCACC	

## Data Availability

The data used in this study are presented in the manuscript. The raw miRNA data obtained from colostrum and mature milk exosomes of the Bamei and Landrace pigs are stored in the NCBI SRA database (BioProject: PRJNA940673). https://dataview.ncbi.nlm.nih.gov/object/PRJNA940673?reviewer=j14pq0i2tpskcak95htmbclass (accessed on 6 February 2024).
